# A case of non‐alcoholic steatohepatitis complicated with severe acute pancreatitis induced by decreased lipoprotein lipase and hepatic triglyceride lipase activity levels in a young Japanese woman

**DOI:** 10.1002/ccr3.1706

**Published:** 2018-07-18

**Authors:** Sawa Minohara, Sung Kwan Bae, Saori Sugiyama, Noriko Shibata, Toshifumi Gushima, Junichi Motoshita, Shinji Shimoda, Atsuko Takagi, Yasuyuki Ikeda, Kazuhiro Takahashi

**Affiliations:** ^1^ The Center for Liver Disease Hamanomachi Hospital Chuo‐ku, Fukuoka Japan; ^2^ Department of Pathology Hamanomachi Hospital Fukuoka Japan; ^3^ Medicine and Biosystemic Science Kyushu University Fukuoka Japan; ^4^ Department of Molecular Pharmacology National Cerebral and Cardiovascular Center Research Institute Osaka Japan

**Keywords:** hepatic triglyceride lipase, hypertriglyceridemia, lipoprotein lipase, non‐alcoholic steatohepatitis, severe acute pancreatitis

## Abstract

We report a case of non‐alcoholic steatohepatitis complicated with acute pancreatitis induced by hypertriglyceridemia in a young Japanese woman. A precise examination of the lipid profile showed decreased lipoprotein lipase (LPL) and hepatic triglyceride lipase activity levels, while the LPL mass was at the minimum level of the normal range.

## 1 INTRODUCTION

Previous studies have shown that hypertriglyceridemic pancreatitis accounts for approximately l%‐13% of all cases of pancreatitis^1^ and 20% of all cases of non‐alcoholic and nonbiliary pancreatitis.^2^ Lipoprotein lipase (LPL), which is one of the key enzymes in the metabolism of triglyceride (TG)‐rich lipoproteins, is produced in fat tissue, skeletal muscle, and heart muscle.^3^ When activated by its cofactor apolipoprotein C‐II (apoC‐II), LPL promotes the hydrolysis of TG, which is transported by chylomicron (CM) and very‐low‐density lipoprotein (VLDL). Patients lacking LPL or apoC‐II develop massive hypertriglyceridemia due to the accumulation of both CM and large VLDL.^4^, ^5^, ^6^ Hepatic triglyceride lipase (HTGL) catalyzes the hydrolysis of TG in the VLDL remnants. HTGL deficiency results in the elevation of cholesterol and TG with the impaired clearance of the VLDL remnants. We herein report a case of non‐alcoholic steatohepatitis (NASH) in a young woman complicated with severe acute pancreatitis (SAP), which was induced by hypertriglyceridemia associated with significantly low activity levels of LPL and HTGL.

## 2 CASE REPORT

**Figure 1:**
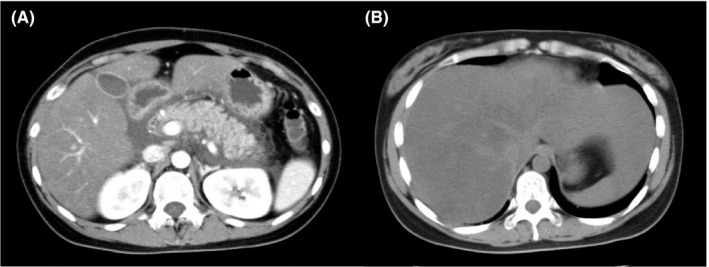
A, Enhanced abdominal CT revealed swelling of the pancreas with surrounding fat stranding and fluid accumulation. B, Plain abdominal CT revealed diffuse and large low‐density areas in the liver, suggestive of moderate to severe fatty liver

**Figure 2:**
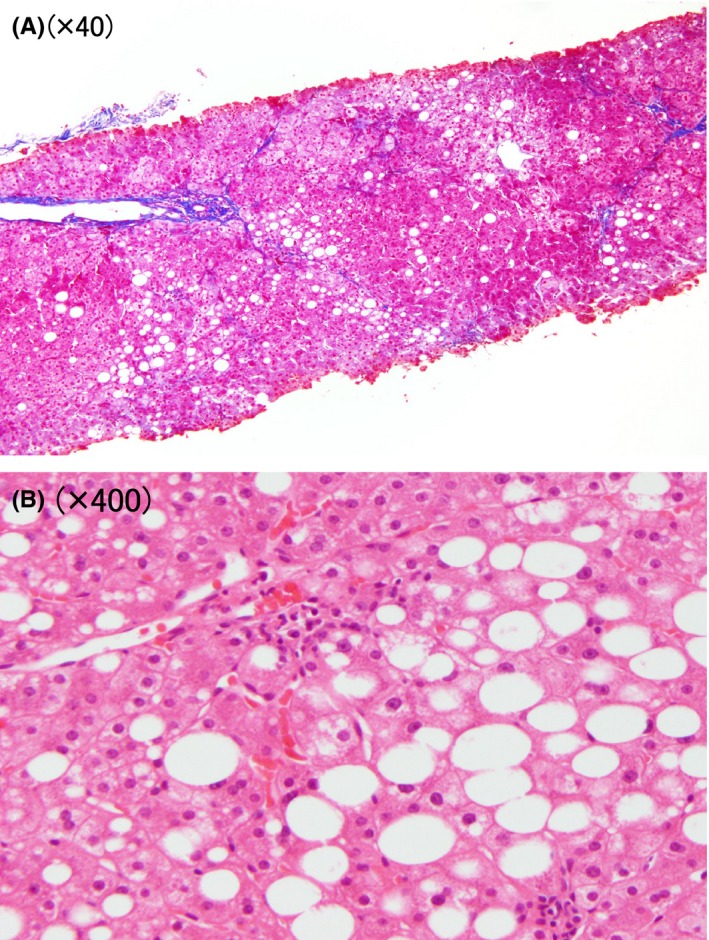
A Liver Biopsy on Day 1008. A, Moderate fibrosis was observed in the low‐power field (Azan‐Mallory stain, x40). B, Higher magnification showed hepatic steatosis, hepatocyte ballooning, and polymorphonuclear cell inflammation (HE staining, ×400)

A 22‐year‐old woman was admitted to our hospital with a two‐day history of intermittent epigastric pain and nausea without any evidence of trauma. Her past medical history showed an episode of hypertriglyceridemia 2 years before admission. One of her cousins had a history of acute pancreatitis, but the details were unclear. She reported that she did not consume alcohol. She had not been taking any drugs, dietary supplements, or herbal medicines. She had a normal constitution (height, 159 cm; weight, 58.8 kg; BMI, 23.26). On examination, she had severe epigastric pain with symptoms of mild tetany. Her blood pressure was 116/80 mm Hg, her heart rate was 72 beats/min, and her body temperature was 36.5°C. Her blood test results (Table 1) were as follows: amylase (AMY), 230 U/L; aspartate aminotransferase (AST), 36 U/L; alanine aminotransferase (ALT), 46 U/L; total cholesterol (T‐Cho), 1225 mg/dL; triglyceride (TG), 8595 mg/dL, low‐density lipoprotein cholesterol (LDL‐C), 58.5 mg/dL; high‐density lipoprotein cholesterol (HDL‐C), 19.5 mg/dL; and serum calcium (Ca), 7.39 mg/dL. Abdominal enhanced CT revealed swelling of the pancreas with surrounding fat stranding and fluid accumulation that resulted in the thickening of the left renal fascia (Figure 1A). Furthermore, plain abdominal CT revealed diffuse and large low‐density areas in the liver, suggestive of moderate to severe fatty liver (Figure 1B). No stones, tumors, or congenital anomalies were found in the bile duct or pancreatic duct by magnetic resonance cholangiopancreatography. Based on these findings, she was diagnosed with severe acute pancreatitis and treatment was initiated with gabexate mesilate (2000 mg/d) and meropenem (1000 mg/d) under fasting conditions.^7^ On day 15, her CRP levels and serum amylase levels normalized and she was observed to have recovered from pancreatitis; thus, the oral intake was started under calorie and fat restriction. The patient had an uneventful clinical course and was discharged on day 24. The day before discharge, her LPL and HTGL levels were examined using postheparin plasma (Table 2). Her LPL activity was lower than the minimal level of the normal range, whereas her LPL mass was almost at the minimal level of the normal range. Similarly, although the HTGL mass was not measured in this study, her HTGL activity was lower than the minimal level of the normal range. After receiving informed consent from the patient and her parents, we performed a genetic analysis to search for an LPL gene mutation. The DNA fragments encoding each of the nine exons of the LPL gene were amplified by a polymerase chain reaction (PCR) and sequenced as previously described.^8^, ^9^ The patient had no specific mutations in exons 1‐9 of the LPL gene. After discharge, bezafibrate (400 mg/d) was started to treat hypertriglyceridemia, and her TG level gradually declined. However, her transaminase level did not normalize. Liver biopsy on day 1008 after the 1st admission showed moderate fibrosis (Figure 2A), and hepatic steatosis and inflammation with hepatocyte ballooning, consistent with a diagnosis of NASH (Non‐alcoholic fatty liver disease activity score,^10^ 3; Matteoni classification,^11^ type 4; Brunt classification,^12^ Grade 1/Stage 2) (Figure 2B). At that point, her serum Wisteria floribunda agglutinin‐positive Mac‐2‐binding protein (WFA^+^‐M2BP) level was 2.33 COI (normal; 0‐0.99). Finally, the patient was treated with fenofibrate (160 mg/d) and omega‐3 fatty acid (2000 mg/d). At the time of this report, she is doing well with normal TG and transaminase levels.

**Table 1:** The laboratory findings

	Case	Reference		Case	Reference
White blood cells (10^3^/μL)	13.4	3.3‐8.6	CRP (mg/dL)	13.3	0‐0.25
Red blood cells (10^4^/μL)	385	435‐555	Glucose (mg/dL)	142	60‐90
Hemoglobin (g/dL)	17.9	13.7‐16.8	HbA1C (%)	4.5	4.3‐5.8
Platelet (10^3^/μL)	163	15.8‐34.8	Total cholesterol (mg/dL)	1225	142‐248
Total protein (g/dL)	6.1	6.6‐8.1	Triglyceride (mg/dL)	8595	40‐234
Albumin (g/dL)	3.6	4.1‐5.1	HDL‐C (mg/dL)	19.5	38‐90
Total bilirubin (mg/dL)	0.27	0.4‐1.5	LDL‐C (mg/dL)	58.5	65‐163
AST (U/L)	46	13‐30	Apo C‐II (mg/dL)	12.3	1.5‐3.8
ALT (U/L)	36	10‐42	IgG (mg/dL)	1205	870‐1700
LDH (U/L)	368	124‐222	IgA (mg/dL)	498	110‐410
Amylase (U/L)	230	33‐120	IgM (mg/dL)	136	46‐260
ALP (IU/L)	208	106‐322	ANA	Negative	<x 40
γGTP (IU/L)	107	13‐64	AMA	Negative	<x 20
BUN (mg/dL)	9	8.0‐22.0	HBsAg	Negative	
Creatinine (mg/dL)	1.3	0.65‐1.07	Anti‐HCV	Negative	
Calcium (mg/dL)	7.3	8.4‐10.2			

ALP, alkaline phosphatase; ALT, alanine aminotransferase; AMA, antimitochondrial antibodies; ANA, antinuclear antibodies; Anti‐HCV, hepatitis C virus antibody; Apo C‐II, apolipoprotein C‐II; AST, aspartate aminotransferase; CRP, C‐reactive protein; HBsAg, hepatitis B surface antigen; HDL‐C, high‐density lipoprotein cholesterol; Ig, immunoglobulin; LDL‐C, low‐density lipoprotein cholesterol; T‐Chol, total cholesterol; γGTP, γ‐glutamyltranspeptidase.

**Table 2:** The LPL and HTGL levels in a postheparin plasma sample

	Patient	Reference
LPL		
Mass (ng/mL)	151	146‐286
Activity (μmol FFA/h/mL)	5.55	7.2‐14.3
HTGL		
Mass (ng/mL)	NT	920‐2858
Activity (μmol FFA/h/mL)	13.5	14.8‐42.6

FFA, free fatty acid; HTGL, hepatic triglyceride lipase; LPL, lipoprotein lipase; NT, not tested.

The LPL activity and immunoreactive LPL mass were determined by subtracting the preheparin plasma values from the postheparin plasma values. The HTGL activity was determined in the same manner.

## 3 DISCUSSION

Hypertriglyceridemia is an established cause of acute pancreatitis. However, the mechanism by which hypertriglyceridemia leads to pancreatitis remains unclear. It is noteworthy that in addition to acute pancreatitis, NASH was also seen in our case. NASH can lead to cirrhosis, which is associated with the risk of progression to portal hypertension and hepatocellular carcinoma. NASH is defined by the presence of hepatic steatosis and inflammation with hepatocyte injury (ballooning) with or without fibrosis.^13^ In our case, a liver biopsy showed hepatocyte ballooning, polymorphonuclear cell inflammation, and moderate fibrosis. These findings were compatible with a pattern of NASH. Furthermore, the WFA^+^‐M2BP level at the time of the liver biopsy was 2.33 COI (normal: 0‐0.99). Nishikawa et al^14^ previously reported that the serum WFA^+^ ‐M2BP level can be a useful marker for assessing the liver histology of patients with NASH. They indicated that the median values in each stage of fibrosis were 0.7 COI in F1, 0.7 COI in F2, 1.2 COI in F3, and 2.4 COI in F4. Based on these results, the possibility that the fibrosis of our patient had already reached an advanced stage cannot be denied. Close monitoring by both liver biopsy and serum fibrosis markers is needed.

Massive hypertriglyceridemia was considered to be the cause of pancreatitis and fatty liver in our case; however, the mechanism that triggered the elevation of the patient's TG level was initially unclear. Based on the episode of hypertriglyceridemia that occurred 2 years before the patient's admission, it is assumed that the patient had chronic hypertriglyceridemia for a number of years. Secondary hypertriglyceridemia caused by factors such as obesity, diabetes, heavy drinking, endocrine disease, nephrotic syndrome, or systemic lupus erythematosus, and drug‐induced secondary hypertriglyceridemia was ruled out. Finally, primary hypertriglyceridemia due to decreased LPL and HTGL activity levels was revealed by a biochemical examination and a search for a gene mutation. Our case exhibited low levels of both LPL and HTGL activity. A literature search revealed only three cases showing reduced levels of both LPL and HTGL activity.^15^, ^16^, ^17^ Fujita et al^17^ reported a case of adolescent hyperlipoproteinemia that was associated with decreased levels of LPL and HTGL activity. The existence of autoantibodies or inhibitors against LPL and HTGL was discussed as a possible explanation for this condition in previous reports^18^, ^19^; however, the exact mechanism has not been clarified. Our case showed no sign of immuno‐dysfunction, and fenofibrate and omega‐3 fatty acid treatment effectively ameliorated the patient's hypertriglyceridemia without any immunosuppressive therapy. Further studies are needed to clarify this rare and important condition.

In our case, the manifestation of SAP gave us the opportunity to identify severe fatty liver. Our experience indicates that when a patient presents with abnormalities of both LPL and HTGL, even if the patient is young and has no history of obesity and diabetes, hypertriglyceridemia can occur and cause NASH silently. In particular, young women, such as the patient in the present case, may face problems during pregnancy. Suga et al^20^ reported the case of a 40‐year‐old Japanese woman with a history of recurrent aggravation of hypertriglyceridemia with LPL deficiency. They indicated that pregnancy seriously exacerbates hypertriglyceridemia and increases the risk of acute pancreatitis in women with LPL deficiency, endangering both the mother and fetus. In that respect, our patient should undergo close long‐term monitoring in order to ensure normal and safe pregnancy.

In conclusion, we reported a case of NASH accompanied by SAP in a young Japanese woman. To the best of our knowledge, this is the first report of NASH caused by decreased levels of LPL and HTGL activity. Abnormalities of both LPL and HTGL can cause hypertriglyceridemia and evoke NASH, even in young patients with no history of obesity and diabetes. We urge clinicians to check the LPL and HTGL levels and dysfunction of patients with atypical hypertriglyceridemia, and not to miss this silent risk of advanced liver fibrosis, especially in young patients.

## AUTHORSHIP

SM: designed the study and wrote the initial draft of the manuscript. SKB: contributed to analysis and interpretation of data, and assisted in the preparation of the manuscript. All other authors have contributed to data collection and interpretation, and critically reviewed the manuscript. All authors approved the final version of the manuscript and agree to be accountable for all aspects of the work in ensuring that questions related to the accuracy or integrity of any part of the work are appropriately investigated and resolved.

## CONFLICT OF INTEREST

The authors declare no conflict of interests in association with this manuscript.
